# 3D printed electrode-microwell system: a novel electrochemical platform for miRNA detection

**DOI:** 10.1007/s00604-025-07190-1

**Published:** 2025-05-01

**Authors:** Panagiota M. Kalligosfyri, Chloe Miller, Stefano Cinti, Bhavik Anil Patel

**Affiliations:** 1 Department of Pharmacy, University of Naples Federico II, 80131 Naples, Italy; 2 Centre for Lifelong Health and School of Applied Sciences, University of Brighton, Brighton, BN2 4GJ UK; 3 Bioelectronics Task Force at University of Naples Federico II, Via Cinthia 21, 80126 Naples, Italy; 4 Sbarro Institute for Cancer Research and Molecular Medicine, Center for Biotechnology, College of Science and Technology, Temple University, Philadelphia, PA 19122 USA

**Keywords:** 3D printing, Electroanalysis, Square wave voltammetry, Conductive PLA, MiRNA, Diagnostics

## Abstract

3D printing has enabled the ability to make creative electrochemical well designs suitable for a wide field of electrochemical sensing. The demand for robust electrochemical systems is particularly high in diagnostics, where the rapid detection of emerging biomarkers associated with severe diseases is critical for rapid medical decision-making. This study is aimed at developing a fully 3D-printed electrochemical sensing device featuring a three-electrode system fabricated from conductive printing materials and incorporating a microwell as the sensing platform. The assay principle of a robust electrochemical screen-printed sensor was adapted for this platform, incorporating a well-structured design to enhance fluid control. This structure ensured the uniform distribution of reagents across the sensing surface, improving the reproducibility and consistency of measurements and enabling the reliable detection of a microRNA target associated with lung cancer. The detection process was based on the hybridization of the target miRNA with an immobilized DNA probe labeled with methylene blue as a redox mediator. The sensor was thoroughly characterized and optimized, achieving a dynamic detection range of 0.001 to 400 nM and a lower limit of detection compared to screen-printed sensors, down to the picomolar level. Furthermore, the sensor demonstrated high selectivity for the target miRNA compared to other miRNA sequences, proving its specificity. These results highlighted the potential of 3D printing technology for the development of sensitive and selective tools for biomarker detection, making it a valuable complementary method in the field of diagnostics.

**Graphical Abstract:**

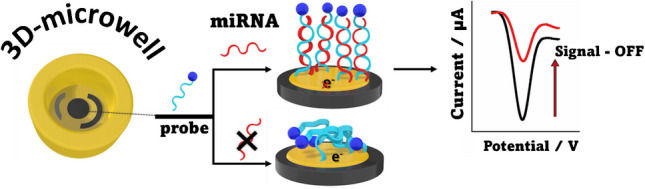

**Supplementary Information:**

The online version contains supplementary material available at 10.1007/s00604-025-07190-1.

## Introduction

In recent years, electrochemistry has benefited from the unique benefits of 3D printing. Traditional electrodes are mainly cylinders or disc geometries, while 3D printing provides the scope to make electrodes of any geometry [1, 2], shape, or size [3]. Fused Filament Fabrication (FFF) is the most widely used 3D printing approach due to the wide array of conductive thermoplastics available (both commercially and made in-house) [4, 5]. FFF printing extrudes a semi-molten thermoplastic onto a heated build platform, using Computer-Aided Design (CAD) software to slice the object, prior printing, into layers and generate a printer-compatible file for precise parameter control [6]. This enables simultaneous printing of conductive and non-conductive thermoplastics, allowing complete electrochemical sensing devices in a single print [7].

By incorporating electrodeposition of materials such as Prussian blue, gold, or copper, it enhances sensitivity and selectivity for analyte detection [8–10]. As a result, FFF printing has facilitated the development of advanced electroanalytical measurement devices, widely applied in biological research [3, 11–13], including sensors designed to interface with biological tissue [14] and multiwell platforms for analyzing biomolecules from organoids [15].

3D printed electrodes enable design flexibility, material efficiency, and miniaturization but face challenges in balancing high-resolution small-scale fabrication with performance. Durability often falls short of conventional electrodes, while conductive and biocompatible materials remain under development. Long-term environmental stability is unproven. Screen-printed electrodes, though cost-effective, suffer from batch variability. Fused Filament Fabrication allows rapid production but demands precise parameter tuning [16–18]. Overcoming these limitations is critical for advancing applications in biosensing systems.

One key area within electroanalytical sensing that 3D printing has shown to be of great promise is the development of microfluidics [19, 20] and lab-on-chip applications [21] and microwells [22–25]. Initially, 3D printing was utilized to make microwells which could serve as a vessel to incorporate electrodes [25]. However, there have been various studies that have utilized the ability to dual print, where conductive carbon thermoplastic electrodes are incorporated within the microwell to make a complete sensing device [7]. Although this approach is highly effective to make sensing devices, it requires the need to dual print, which is not always accessible. Therefore, in this study, we developed a simple electrochemical well design that allows the ability to click in electrodes within the microwell. To demonstrate its advantageous application, miRNA diagnostics has been considered as the model diagnostics application. Circulating miRNAs show great promise for diagnosing and prognosing various diseases, including cancer, cardiovascular, and neurodegenerative conditions [26]. However, gold-standard quantification methods like next-generation sequencing (NGS), CRISPR-based systems, and droplet digital PCR are costly, require specialized personnel, and involve lengthy processing times [27]. These challenges can be addressed through the development of new analytical sensing tools, which are designed to be cost-effective, portable, sensitive, and easy to use. It should be noted that the adoption of 3D printing might provide the ability to use varying materials for electrodes manufacturing and importantly modify them prior to incorporating within the microwell, for the chosen application. With regard to existing electrochemical sensors and biosensors, various approaches towards miRNA detection have highlighted their potential, particularly for their portability and simplicity. These platforms use diverse assay configurations, such as intercalative labeling, enzyme-driven signal amplification, magnetic bead integration, and functionalized nanomaterials usually conducted on paper-based microfluidics making them highly versatile and efficient [28–31]. Recent advancements in 3D printing have facilitated the development of 3D printed microfluidics, which were employed for sample preparation, purification, and preconcentration [32], as well as in 3D printed droplet-based PCR devices [33].

In the context of electrochemical sensors, 3D printing enables rapid and customized designs that improve assay specificity, enable surface modifications, and allow the use of flexible materials. Compared to conventional screen-printed or commercial electrodes, 3D-printed sensors provide enhanced liquid handling and facilitate the integration of reusable components, supporting sustainable and cost-effective analytical platforms. Additionally, the compatibility of 3D printing with miniaturized electronics, enabled by conductive filaments, further enhances the potential for portable and point-of-care applications [5, 17, 34].

To meet the growing demand for customizable and high-performance electrochemical devices [17], we designed and fabricated a fully FFF 3D printed electrochemical sensing well that offers the ability to click-in electrodes. This click-in configuration uses the advantages of 3D printing to create a versatile, adaptable, and reusable sensing platform tailored for advanced analytical applications. The detection mechanism builds upon established principles from robust screen-printed electrochemical sensors [35–37] developed for miRNA analysis. In this assay, miRNA is detected through hybridization with a specific DNA probe that is labeled with methylene blue (MB). Methylene blue functions as a redox mediator, facilitating electron transfer between the hybridized probe and the working electrode. This interaction allows the detection of the target miRNA by translating the hybridization event into a measurable current response. Methylene blue is widely employed as a labeling agent in electrochemical hybridization assays due to its ability to enhance the sensitivity and efficiency of detection. It has been successfully used for labeling aptamers and single-stranded DNA probes in the detection of miRNAs, nucleic acids, and even exosomes [38–40].

By introducing an interdisciplinary approach that combines 3D printing technology with miRNA electrochemical sensing, we have created an innovative platform for biomarker detection. The miRNA detection principle relies on the hybridization of the target miRNA with a specific DNA probe immobilized on the gold-modified surface of the 3D printed electrode. This probe is labeled with methylene blue, a redox mediator. Upon target recognition, a decrease in the signal response occurs, functioning as a signal-OFF detection system. The 3D printed sensor was evaluated for its analytical performance and was successfully applied to commercial serum samples, proving its potential for broader diagnostic applications.

## Experimental section

Carbon black/polylactic acid (CB/PLA) filament (Proto Pasta, purchased from Filaprint, UK) was used for the fabrication of the 3D printed electrodes. All chemicals were of analytical grade, and the redox probe solution was prepared with 1 M potassium chloride (KCl) in deionized water. 5 mM gold chloride trihydrate (HAuCl_4_.3H_2_O) was prepared in deionized water. Ferricyanide (Fe (CN)_6_^3−/4−^) and HAuCl_4_.3H_2_O were purchased from Merck (Gillingham, United Kingdom). The methylene blue (MB) tagged DNA probe (5′- Thiol-C6 AAG AGC CAG TGG TGA AAC ACT G Atto-MB-3′) (anti-miRNA-4676), designed to selectively target miR-4676, along with the target miR-4676 sequence (5′-cacuguuucaccacuggcucuu-3') and the control sequences used in the selectivity study, was obtained from Metabion GmbH (Steinkirchen, Germany). All electrochemical measurements were conducted using a MultiPalmSens4 multi-channel potentiostat (PalmSens, Utrecht, Netherlands), connected to a laptop running PSTrace 5.10 software. Square Wave Voltammetry (SWV) for miRNA detection was carried out over a potential range of 0.05 to − 0.6 V, with an amplitude of 0.01 V and a frequency of 50 Hz.

### Production of 3D printed PLA microwell

The designs for both the three-electrode system and the microwell were designed using Solidworks computer-aided design software and are shown in Fig. S1 and Fig. S2, which were based on the patterning observed in a screen-printed electrode. The working electrode was 4 mm in diameter. Both the reference and counter electrodes were curved but were 1.5 mm in width. The reference electrode was 5 mm in length, and the counter electrode was 12 mm in length. All electrodes were 3 mm in depth. For the well in which the electrodes were clicked in, the diameter was 20 mm, and the depth was 10 mm. The well was designed to hold 500 µL of liquid (Figure S1).

### Production of the click-in 3D printed CB/PLA electrodes

CB/PLA filament was used to make all three electrodes; working, counter, and reference were printed in accordance with the design of the microwell with the measurements taken from a screen-printed electrode, as detailed above. All electrodes were printed using a Flash Forge Creator Pro printer, where the extruder was set to 230 °C, and the print bed was 50 °C. The print layer thickness was 0.1 mm. These parameters were set based on previous studies conducted [41, 42]. The reference electrode was painted with silver paint and left in bleach for 90 s to chlorinate the reference surface, as shown in a previous study [43]. The electrodes were then clicked into the microwell casing. The ohmic connection to each of the electrodes was made by embedding the silver wire into the electrode surface.

### Electrochemical characterization

The electrochemical characterization experiments were conducted with a CHI760E potentiostat (CH instruments, Texas). Before conducting electrochemical experiments, the microwell working electrode was electrochemically pretreated using 0.5 M sodium hydroxide by holding the potential at 1.4 V, then at − 1.0 V for 200 s each way *vs.* the silver chlorinated reference [43]. Cyclic voltammetry measurements were conducted in 5 mM ferricyanide (Fe (CN)_6_^3−/4−^) solution in 1 M KCl with the potential window of − 0.2 to 0.6 V *vs.* silver chlorinated reference.

### Development of miRNA sensor

After initial Fe (CN)_6_^3−/4−^ measurements, 5 mM HAuCl_4_.3H_2_O was electrodeposited onto the surface of the working electrode via chronoamperometry at − 0.5 V for 600 s *vs.* silver chlorinated reference. The concentration was chosen based on previously published work [44]. The electrodes were cleaned in distilled water, and Fe (CN)_6_^3−/4−^ measurements were conducted under the same conditions after the deposition of gold. The methylene blue (MB)-labeled DNA probe (anti-miR-4676), containing a thiol group, was reduced in the presence of tris(2-carboxyethyl) phosphine (TCEP) and immobilized onto the gold-deposited working electrode through gold-sulfur interactions, as reported previously [35, 37]. A 100 µL solution of 50 nM reduced probe was incubated for 1 h in a humidity chamber to facilitate immobilization. After incubation, the electrodes were washed with 1 mL of water (2 × 500 µL). The microwell was gently dried avoiding contact with the electrodes, ensuring it did not dry completely. Next, 100 µL of 2 mM 6-mercapto-hexanol, used as a blocking solution, was added. A 30-min incubation step was followed in the humidity chamber, and the washing step was repeated. The electrodes were then stabilized for 30 min before usage.

### Calibration curve of miRNA

After stabilization, the appropriate volume of target miRNA (with concentrations ranging from 0.001 to 400 nM) or phosphate-buffered saline (PBS), i.e., the blank sample, was added to the microwells, and detection was carried out within 30 min. The electrochemical sensor responses were recorded using square wave voltammetry (SWV) over a potential range of 0.05 to − 0.6 V, with an amplitude of 0.01 V and a frequency of 50 Hz. The results were analyzed as the percentage change in signal, calculated by comparing the current intensity between the blank sample (absence of target) and the presence of the miRNA target.

### Detection of miRNA in commercial serum

For miRNA detection, commercial serum was used as the sample matrix. The analysis was carried out in 1% diluted serum samples spiked with miRNA target at concentrations ranging from 0.02 to 100 nM. These spiked samples were incubated on the prepared miRNA 3D printed sensor. Following a 30-min incubation, the sensor response was recorded using SWV as previously described. The results were analyzed by calculating the percentage change in signal, determined by comparing the current intensity of the blank sample (in the absence of the target) with that obtained in the presence of the miRNA target.

## Results and discussion

### Fabrication of the click-in 3D printed electrode microwell

**Fig. 1:**
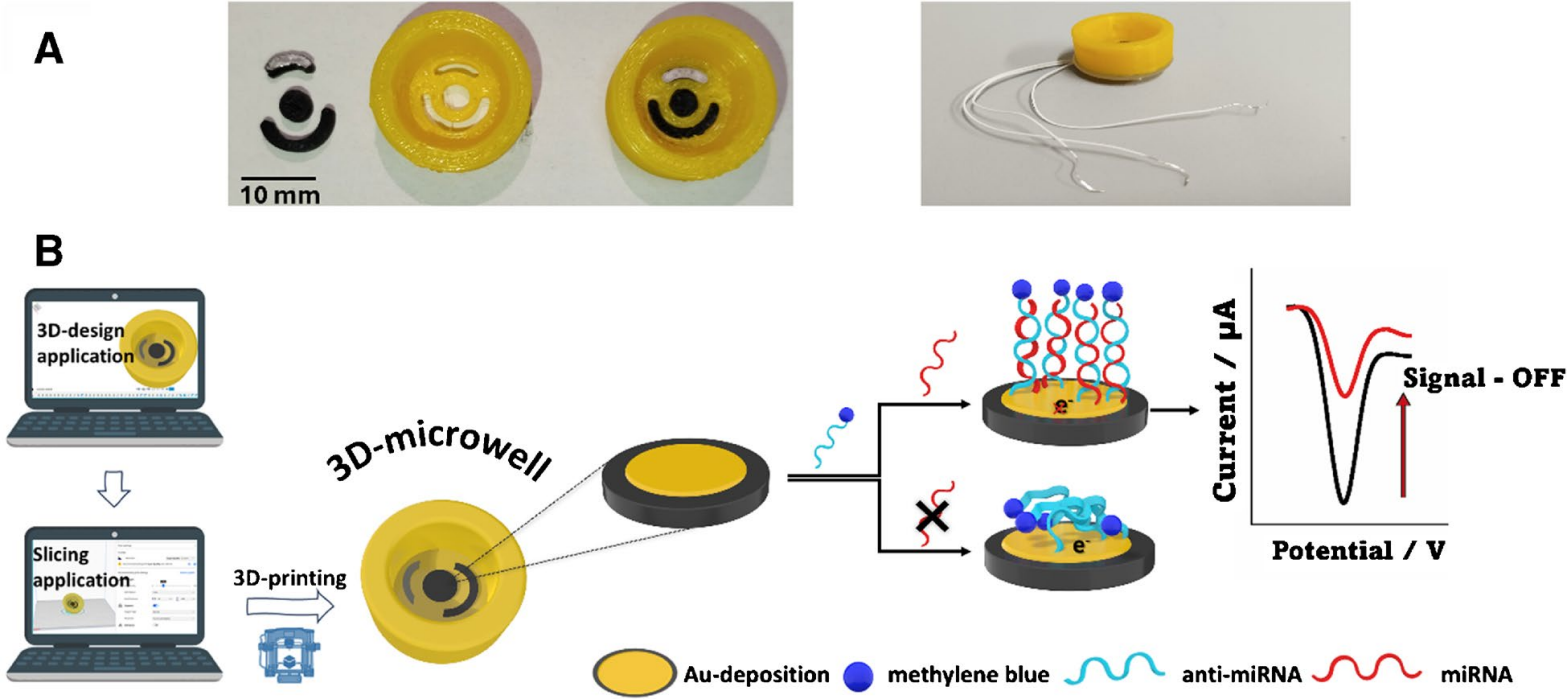
**A** Production of FFF microwell with click-in electrodes. The figure shows the printed three-electrode setup, with a silver chlorinated reference and the well as printed. The electrodes are clicked into the microwell, with the ohmic connection produced through the embedding of a platinum wire for connection to the potentiostat. **B** Each stage of the process is illustrated, from initial design and fabrication to the assay’s detection principle

Figure 1A shows a photograph of the individual electrodes and the microwell before the electrodes are clicked in to make the complete sensing device. Once the electrodes were clicked in and the connections were made and glued, the device became leak proof. The well structure in the sensor design plays a significant role in enhancing fluid control by ensuring uniform distribution of reagents across the sensing surface, which in turn improves the reproducibility and consistency of the measurements.

Sealing the electrodes was a critical step in ensuring the fabrication of a leakproof device, particularly since the infill pattern used during 3D printing was not of 100% density, which could result in minor leakages. To address this, and to ensure a tight seal around the areas where the electrodes were clicked in, a soldering iron was carefully applied around the edges of the device. This process allowed the plastic to melt slightly and close any small gaps or holes that might compromise the integrity of the design. Following this, the entire back of the device was coated with glue, further sealing the structure and enhancing its leakproof performance.

This approach proved to be highly reliable, as the 3D printing method and materials used exhibited minimal variation across consecutive prints. In cases where inconsistencies were observed—either in the electrode dimensions or casing—they were identified and discarded. The leakproof quality of the assembled device was verified by testing with deionized water prior to use in actual experiments, confirming that the electrodes fit correctly and that no leakage occurred.

Concerning the conductivity of thermoplastic filaments used for the fabrication of 3D printed electrodes, previous studies have reported that carbon-based materials such as multi-walled carbon nanotubes and carbon black—specifically the Proto Pasta filament used in this study—exhibit relatively high resistivity values of 33.7 ± 3.7 kΩ·m and 4.0 ± 0.3 kΩ·m, respectively [45, 46], with resistivity being inversely proportional to conductivity. Therefore, among the two, carbon black demonstrates significantly higher conductivity due to its lower resistivity. Based on these findings, carbon black was selected for this work due to its comparatively lower resistivity, wide availability in filament form, and cost-effectiveness [47].

The microwell structure provides several advantages: it confines the sensing material within a defined area, preventing sample loss and ensuring efficient analyte interaction. This design enhances signal stability, minimizes reagent consumption, and makes the device highly suitable for biological applications. Additionally, the leak-proof feature ensures reliability in measurements requiring longer incubation times while maintaining a low-cost and scalable fabrication process.

Due to the nature of the experiment, where a specific DNA probe is immobilized, these devices are designed for single use. Once the assay is conducted, the device was not reused. Therefore, long-term stability is not a critical factor for this study. The devices can be printed and stored in the laboratory until the assay is performed. During the probe immobilization, stabilization, and miRNA detection steps, the electrodes were stored in a humidity chamber for the various incubation times required, as outlined in the method section above. The assay principle of the 3D printed electrochemical microwell sensor relies on detecting the miRNA target using a methylene blue (MB)-labeled specific DNA probe, referred to as anti-miRNA. The thiolated DNA probe is immobilized on the gold-coated working electrode through gold-sulfur interactions. In the absence of the miRNA target, the MB label on the probe facilitates electron exchange with the working electrode surface. However, when the miRNA target is present, it hybridizes with the probe to form a short RNA–DNA heteroduplex. This recognition event reduces the number of electrons transferred to the electrode surface, leading to a decrease in the current response. Consequently, the sensor operates as a signal-OFF electrochemical platform, where higher target concentrations result in lower recorded current responses. In Fig. 1B, every step of the process is illustrated from the 3D design and printing to the previously described sensor’s detection principle.

### Characterization of microwells through gold electrodeposition

Commercially available 3D printed thermoplastic filaments typically exhibit a high thermoplastic-to-carbon content, making them unsuitable for direct electrochemical applications [48–50]. Various post-treatment methods are employed to remove thermoplastic residues from the working electrode surface, including thermal treatment, electrochemical treatment with sodium hydroxide or PBS, solvent treatment, and biological approaches [11]. The removal of thermoplastic via saponification [51] allows for more electroactive carbon sites to be available on the surface, enhancing the electrode’s electrochemical performance.

**Fig. 2:**
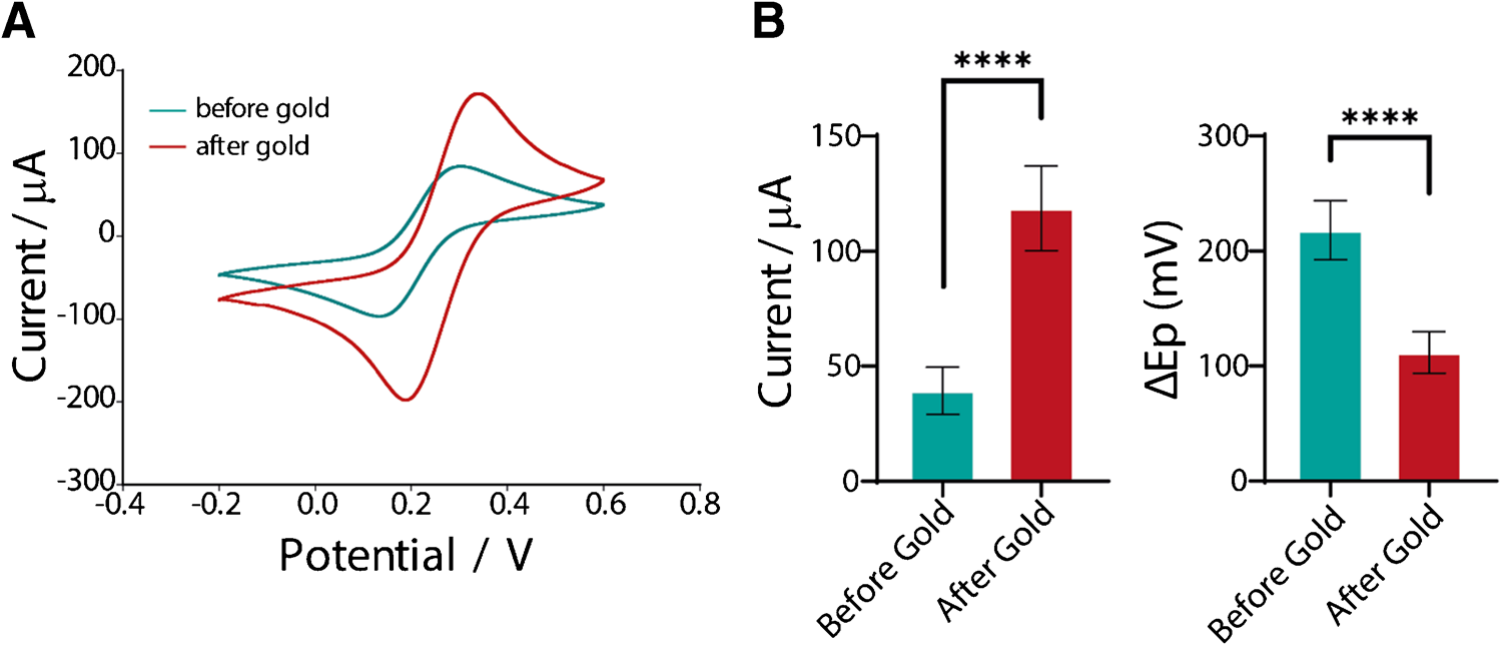
Characterization of microwell for gold deposition using ferricyanide. **A** Cyclic voltammograms of 5 mM ferricyanide in 1 M KCl at 0.05 V s^−1^ before (black line) and after (red line) gold electrodeposition. **B** Histograms of current and ∆Ep values before and after gold deposition. Data shown as mean ± SD, where *n* = 6

In this study, electrochemical pretreatment using sodium hydroxide was conducted to increase the number of active sites available for gold deposition, ultimately improving the sensitivity of the electrochemical sensing device. Fe (CN)_6_^3−/4−^ was chosen as an inner sphere redox probe due to the ability to detect impurities on the surface, which can negatively influence electron transfer [52]. HAuCl_4_.3H_2_O (gold) addition to the surface of electrochemical sensors, whether through electrodeposition or drop cast as shown in previous literature, is mainly of benefit to improve the electrochemical behavior as well as being a base to produce immune and DNA sensors, due to the specific binding between the surface and the biomolecules [53, 54]. Figure 2A shows cyclic voltammograms for the inner sphere probe, showing clear differences before and after the deposition of gold on the microwell surfaces. Figure 2B looks at the comparison between the current densities as well as the ∆Ep values.

The current values for before gold deposition are low, suggesting that without the presence of gold, there are minimal sites present on the surface for an enhanced performance. This is also reflected in a large ∆Ep value. After gold deposition, however, there is an increase in current and a decrease in ∆Ep. This suggests that the use of a metal provides better electron transfer kinetics as the gold acts as an electron facilitator for the conductive sites within the electrode. This can also be seen in previous publications for the addition of gold to the surface of 3D printed electrochemical sensing devices [55]. This therefore provides an improved electrochemical performance in preparation for biological modification.

### Optimization study of the anti-miRNA probe concentration

**Fig. 3:**
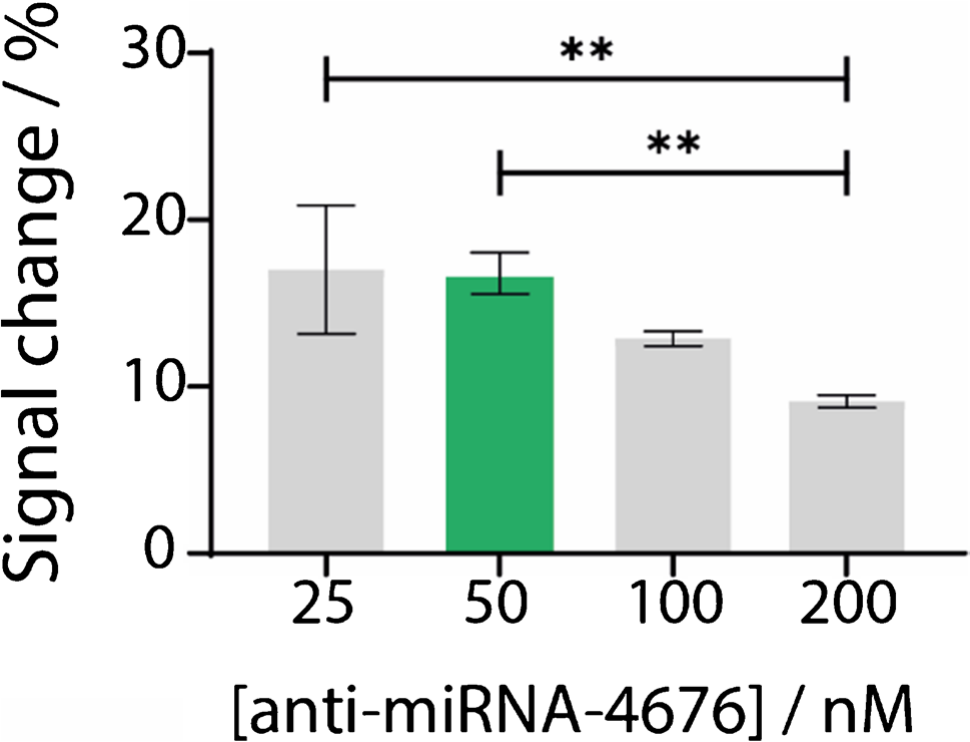
Anti-miRNA probe optimization study. Various probe concentrations ranging from 25 to 200 nM were tested in the presence of 20 nM of target miRNA-4676. Histograms were plotted for each probe concentration against the corresponding signal change %. Data are shown as mean ± SD, *n* = 3

To optimize the immobilization of the DNA probe on the gold-deposited working electrode—a critical factor for target recognition—various concentrations of the anti-miR-4676 specific probe were evaluated. miR-4676 was selected as a model target due to its relevance to the study and its established association with the diagnosis of several diseases such as lung and gastric cancers [56, 57]. Specifically, four concentrations (25, 50, 100, and 200 nM) were tested in the presence of 20 nM of the target miRNA-4676. The aim of this study was to identify the concentration that achieved the highest percentage of signal change while maintaining optimal reproducibility, as indicated by standard deviation. As shown in Fig. 3, the optimal concentration was determined to be 50 nM, which provided the highest signal change percentage and the most consistent results.

### Analytical performance of the 3D printed electrochemical microwell sensor

**Fig. 4:**
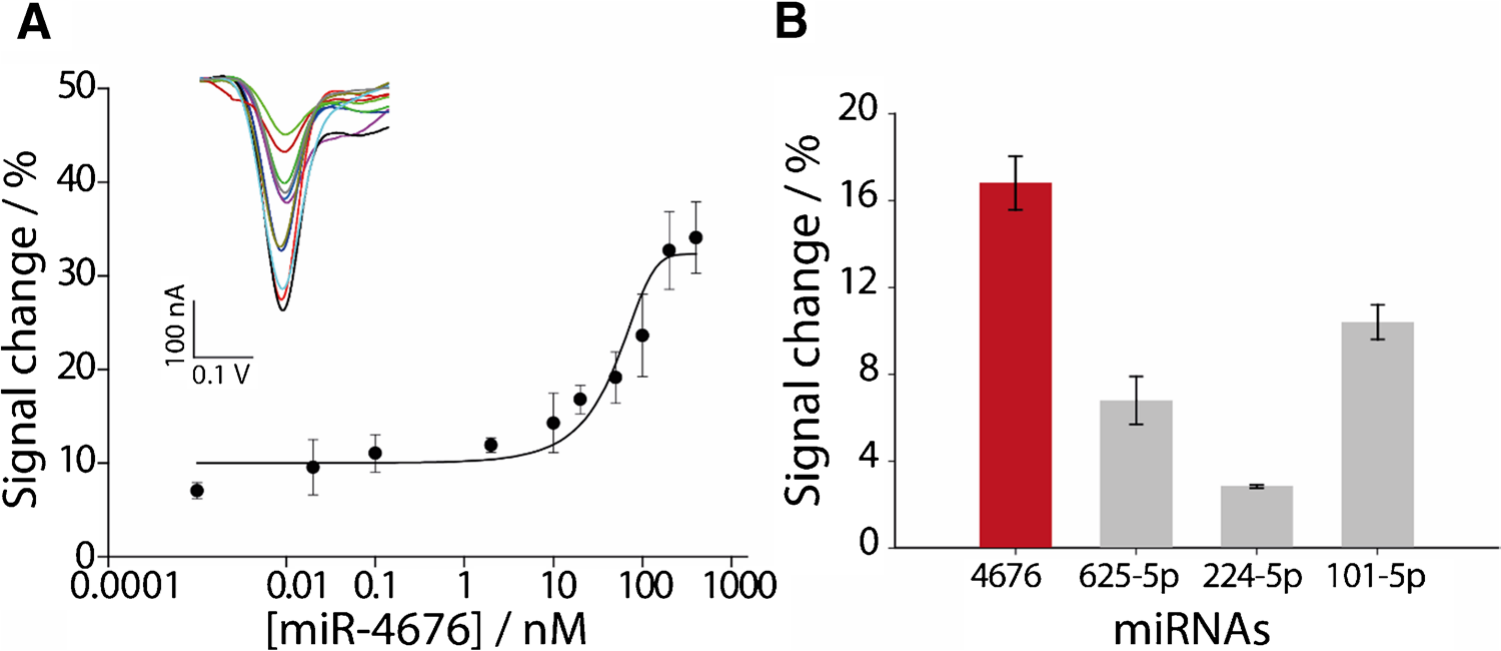
**A** Calibration curve obtained in PBS with increasing concentrations of miR-4676, ranging from 0.001 to 400 nM. The inset shows the SWV curves corresponding to each concentration. The blank sample is represented by the black line. SWV parameters: teq: 5 s; Estart: 0.1 V; Estep: 0.001 V; amplitude: 0.01 V; frequency: 50 Hz. **B** A selectivity study of 50 nM anti-miR-4676 specific DNA probe was performed in the presence of 20 nM of the target mir-4676 and 20 nM of three interferent miRNAs, namely: mir-625-5p, mir-224-5p, and mir-101-5p. Data are shown as mean ± SD, *n* = 3

The analytical performance of the optimized 3D printed electrochemical sensor was assessed by constructing a calibration curve in a buffer solution. The miRNA-4676 target was chosen as a biomarker for its clinical significance, particularly in diagnosing and monitoring non-small cell lung cancer, as part of a validated microRNA signature from a recent clinical trial [56]. The calibration curve was developed using a range of miRNA-4676 concentrations spanning from 0.001 to 400 nM. As illustrated in Fig. 4A, the sensor response exhibited a sigmoidal correlation between the percentage signal change and the logarithmic concentration of miRNA at the nanomolar level.

The limit of detection (LOD), determined from the lower linear range between 0.001 and 2 nM with an equation of *y* = 11.58 + 1.48*x* (*R*^2^ = 0.98) was calculated to be as low as 1 pM, a comparable value to other reported works [31, 35, 36, 58] for miRNA detection (Table S1). These methods allow for a comprehensive comparison of the fully 3D printed sensor with various reported electrodes, including commercial gold screen-printed electrodes (SPEs), commercial carbon-based SPEs, and hand-made SPEs. Additionally, these methods often involve long protocols and multiple working electrode modification steps, such as enzyme immobilization, gold nanoparticle drop casting, or polydopamine-gold complexes. In summary, the fully 3D printed sensor demonstrates similar analytical performance at the pM level, highlighting its potential for use in various applications.

The repeatability of the sensor was evaluated by measuring the relative standard deviation (RSD%). The fully 3D printed electrochemical platform demonstrated an RSD% below 22%, confirming its reproducibility and suitability for practical applications.

To assess the specificity of the 3D-printed microwell sensor, its selectivity was evaluated in the presence of potentially interfering miRNAs, including miR-625-5p (sequence: 5′-agggggaaguucuauagucc-3′), miR-224-5p (sequence: 5′-ucaagcacuagugguuccguuua-3′), and miR-101-5p (sequence: 5′-caguuaucacagugcugaugcu-3′). The optimized concentration of the specific DNA probe employed in this study was 50 nM, while the target miRNA (miRNA-4676), fully complementary to the probe, was tested at a concentration of 20 nM. Interfering miRNAs were likewise assessed at a concentration of 20 nM, and their responses were compared to that of the fully complementary target. The presence of these non-complementary sequences resulted in signal deviations of less than 12% (Fig. 4B), thereby demonstrating the high specificity of the sensor and its robust ability to selectively detect the target miRNA.

To enable the integration of our proposed device into real clinical applications, several strategies should be considered to further enhance its specificity. Future goals should focus on improving the system’s specificity using a variety of approaches. The hybridization buffer plays a crucial role, particularly regarding the sodium chloride concentration [59], which helps maintain the appropriate ionic strength during the hybridization step, facilitating the formation of more stable hybrids between the DNA probe and the miRNA target. Incorporating additional washing steps could further enhance specificity by reducing non-specific binding, although this may increase total assay time. Alternatively, washing steps can be minimized by adding surfactants, such as Tween-20, to the hybridization buffer, as these are known to reduce non-specific interactions. Furthermore, the use of peptide nucleic acid (PNA) probes offers a distinct advantage, as PNAs exhibit superior affinity and specificity for miRNA compared to DNA probes, allowing for the use of shorter probes [60, 61].

Notably, in the case of our proposed fully 3D printed sensor, washing steps are particularly feasible due to the robustness of the device, which utilizes gold deposition rather than gold nanoparticles or paper-based materials that are more sensitive to extensive washing steps.

### Serum analysis

**Fig. 5:**
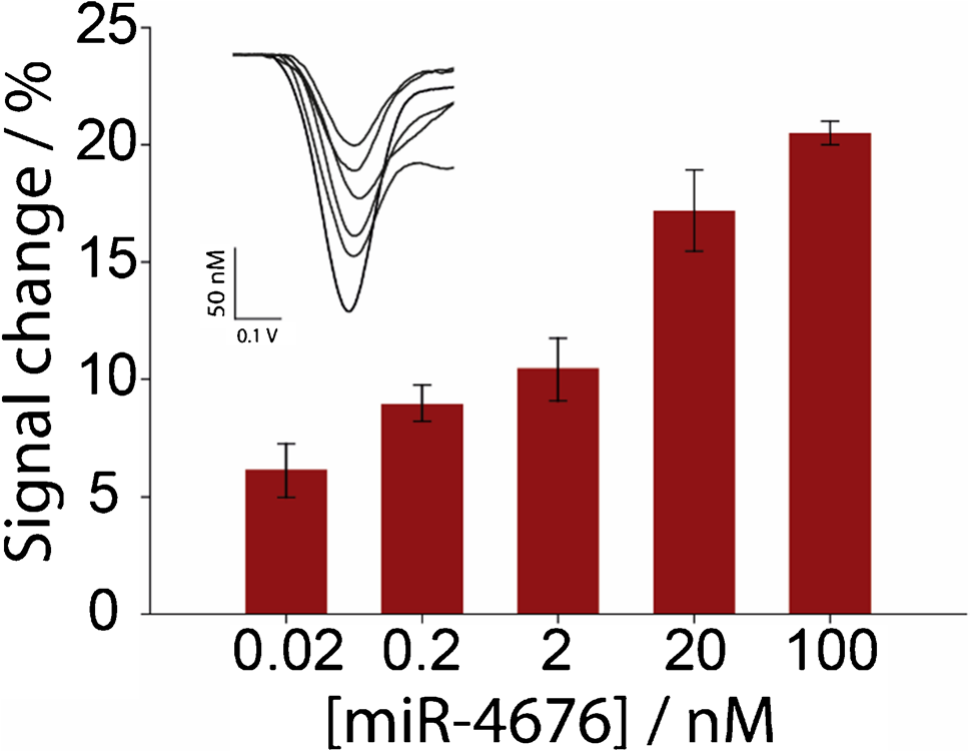
Histograms showing the signal change percentage for 1% spiked commercial serum with miRNA target concentration ranging from 0.02 to 100 nM. The inset shows the SWV curves corresponding to each concentration. Data are shown as mean ± SD, *n* = 3

Following the optimization and analytical characterization of the fully 3D printed electrochemical sensor, commercial serum samples were systematically analyzed by spiking them with varying concentrations of miRNA, ranging from 0.02 to 100 nM. The sensor was applied to 1% diluted commercial serum, which was spiked with five distinct miRNA concentrations: 0.02, 0.2, 2, 20, and 100 nM (Fig. 5).

The results demonstrated the sensor’s effective performance in detecting miRNA within complex biological matrices, underscoring its potential for practical applications in real biological samples. As depicted in the figure, the percentage change in signal varied across the different spiking levels, reflecting the sensor’s sensitivity to miRNA concentrations in serum. Moving forward, we will focus on spiking experiments and the construction of calibration curves using real biological samples to further assess and optimize the sensor’s accuracy and overall reliability in clinical settings.

## Conclusions

This study introduces a novel design, construction, and application of a fully FFF 3D printed electrochemical sensing device for biomolecule detection. The device forms a reusable microwell component and a three-electrode system that can be easily assembled. With an estimated cost of 30 p, which is inclusive of the printing as well as wires for ohmic connections, the proposed device demonstrates that by harnessing the versatility of printing materials, innovative design possibilities, and the customizability of 3D printing, robust and cost-effective alternatives can be developed for electrochemical sensing applications.

To our knowledge, this is the first fully 3D printed device designed for microRNA detection. The device was successfully applied to the analysis of commercial serum samples, highlighting its potential for broader diagnostic applications. The sensor’s limit of detection was found to be as low as 1 pM, a value comparable to other reported methods for miRNA detection (Table S1). In fact, the sensor demonstrated similar analytical performance, at the picomolar level, with studies utilizing commercially available and hand-made screen-printed or bulk electrodes, which have been modified with various approaches such as enzymes, polydopamine, nanoparticles, or gold deposition (like in our study).

Furthermore, the incorporation of a gold-deposition process enables universal modification, allowing the device to be further functionalized and used for the immobilization of various biomolecules, including aptamers and antibodies [34, 62]. This adaptability highlights the developed sensing device as a promising platform for advanced diagnostic solutions in the biomedical field.

While 3D printed electrodes offer significant advantages, such as design flexibility, material efficiency, and the potential for miniaturization, there are several challenges that still need to be addressed. One of the primary concerns is the resolution of the printed electrodes, as achieving high precision at smaller scales can be difficult, which may affect their performance and reliability. Additionally, the mechanical properties of the printed materials may not always match those of conventional electrode materials, potentially limiting their durability and performance in certain applications. Another challenge is the need for appropriate materials that can offer the necessary conductivity and biocompatibility, particularly for applications in biomedical fields. While there has been progress in developing conductive filaments and biocompatible materials, achieving the right balance between these properties is still a work in progress [16–18]. Moreover, the long-term stability of 3D printed electrodes in various environments, such as in vivo conditions or harsh industrial settings, remains a concern.

In comparison, SPEs, while widely used due to their low cost and simplicity, face challenges in terms of reproducibility. The manufacturing process can lead to variability between batches, affecting the consistency and reliability of the electrodes. This lack of reproducibility, driven by factors like ink formulation, substrate quality, and printing conditions, can significantly impact performance, especially in applications where high precision is required [16]. Finally, while FFF printing offers rapid and cost-effective production, it still requires careful optimization of printing parameters, such as temperature, speed, and filament choice, to ensure consistent electrode quality. Overcoming these challenges will be key to realizing the full potential of 3D printed electrodes in various applications.

Moving forward, future work will focus on investigating the fully 3D printed device’s accuracy and overall reliability for clinical diagnostics by utilizing real biological samples. The integration of this sensor into clinical settings could significantly enhance diagnostics and decision-making processes by providing a reliable, cost-effective, and customizable sensing platform for detecting critical biomolecules such as miRNAs.

## Supplementary Information

Below is the link to the electronic supplementary material.

Supplementary file1 (DOCX 663 KB)

## Data Availability

No datasets were generated or analysed during the current study.
