# Supplementary material for: 3D printed electrode-microwell system: a novel electrochemical platform for miRNA detection
Source: Mikrochim Acta. 2025 May 1;192(5):330. doi: 10.1007/s00604-025-07190-1 (PMC12045820; doi:10.1007/s00604-025-07190-1)
Supplement: Supplementary file 1 — Supplementary file1 (DOCX 663 KB) [file 604_2025_7190_MOESM1_ESM.docx]

**Supplementary material**

**3D printed electrode-microwell system: a novel electrochemical platform for miRNA detection**

**Panagiota M. Kalligosfyri^1,+^, Chloe Miller^2,+^, Stefano Cinti^1,3,4,*^ , Bhavik Anil Patel^2,*^**

^1^Department of Pharmacy, University of Naples Federico II, 80131 Naples, Italy

^2^Centre for Lifelong Health  and  School of Applied Sciences, University of Brighton, Brighton BN2 4GJ, United Kingdom

^3^Bioelectronics Task Force at University of Naples Federico II, Via Cinthia 21, Naples 80126, Italy

^4^Sbarro Institute for Cancer Research and Molecular Medicine, Center for Biotechnology, College of Science and Technology, Temple University, Philadelphia, Pennsylvania 19122, United States of America

+These authors contributed equally

*Corresponding authors: [stefano.cinti@unina.it](mailto:stefano.cinti@unina.it), [b.a.patel@brighton.ac.uk](mailto:b.a.patel@brighton.ac.uk)

**Dimensions and configuration of the 3D printed microwell and 3D printed three electrode system**


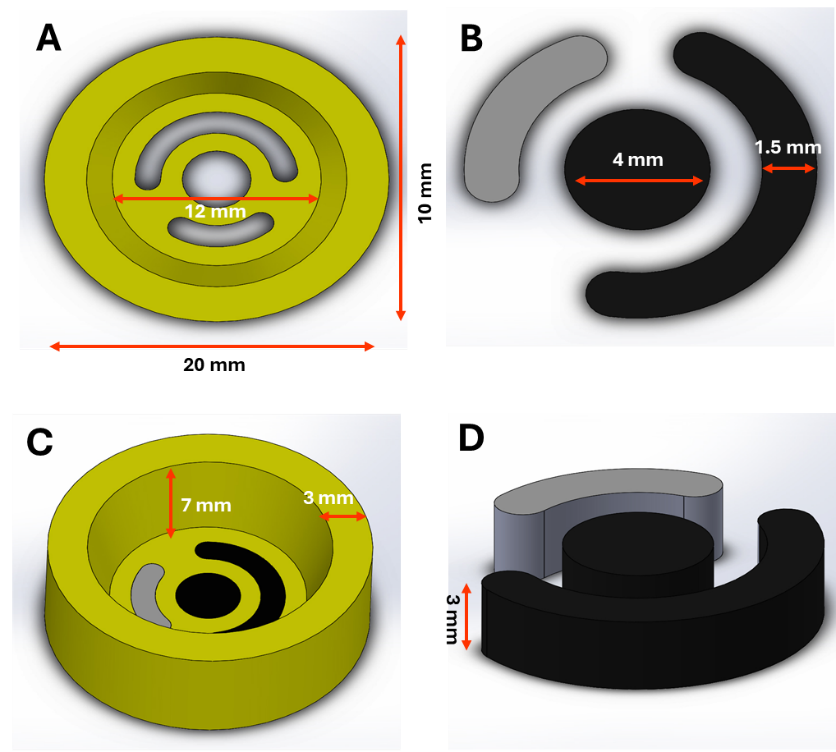


**Figure S1.** CAD designs of the FFF 3D printed microwell and the 3D printed three electrode system. (A) 2D design of the microwell containing the size dimensions. (B) 2D design of the three electrode system containing the working, reference (silver) and counter electrodes with respective dimensions. (C) 3D view of the microwell containing the three electrode system. (D) 3D view of the three electrode system to show the depth of the electrodes in the microwell.


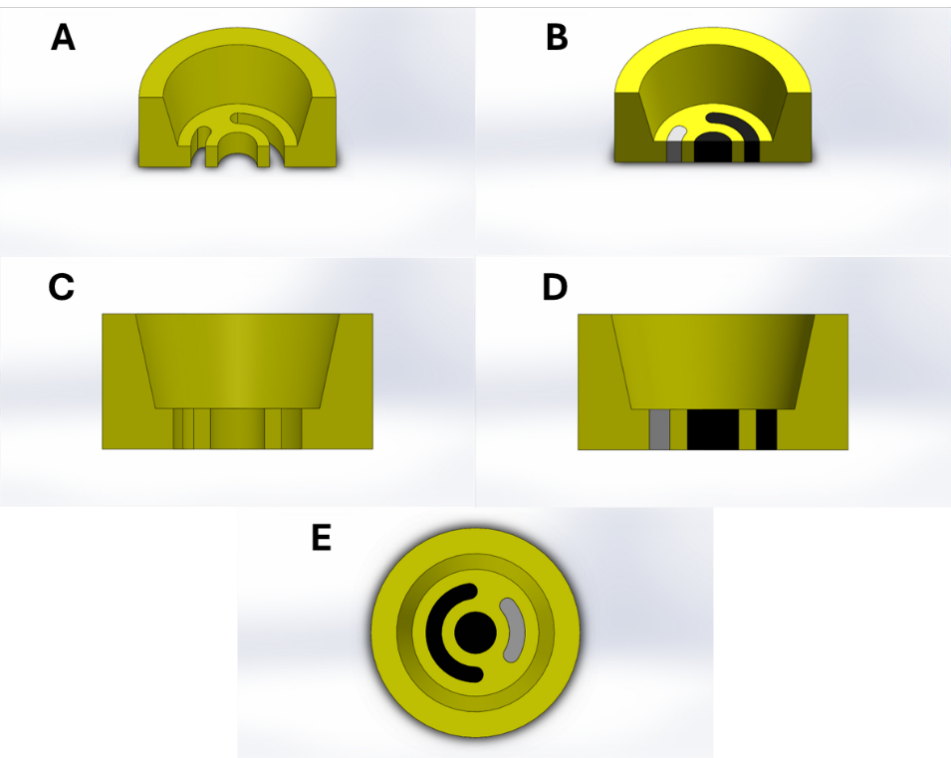


**Figure S2.** CAD Designs of the FFF 3D printed microwell from alternative viewpoints. (A) Side view of the microwell detailing the positioning of the three electrodes to be clicked in. (B) Side view the microwell with the three electrodes clicked in to show equal positioning of the electrodes. (C) 2D side view of the microwell to show the well in relation to where the electrodes are clicked in. (D) 2D side view of the microwell with the electrodes clicked in to show depth of electrodes in the well. (E) Complete CAD design of microwell with electrodes clicked in: reference (silver), working (middle) and counter.

**Table S1:** Various reported electrochemical sensors for miRNA detection, utilizing diverse surface modifications and/or electrode fabrication techniques.

| **Electrodes** | **WE modification** | **Dynamic range / nM** | **Linear range / nM** | **LOD / nM** | **Ref.** |
| --- | --- | --- | --- | --- | --- |
| Personal glucose meter (PGM) | Enzyme | 0.010-100 | 0.010-100 | 0.001 | 31 |
| Commercial gold screen-printed | N/A | 0.01-1000 | N/A | 1 | 35 |
| Screen-printed | AuNPs | 0.01- 500 | N/A | 0.15 | 36 |
| Commercial  screen-printed | Polydopamine-gold composite | N/P | N/P | 0.00026 | 59 |
| Fully 3D printed | Au-deposition | 0.001-400 | 0.001 – 2 | 0.001 | This work |

Ref.: References, WE: Working electrode, LOD: Limit of detection, N/P: Not provided, N/A: Not applied, AuNPs: Gold nanoparticles, Au: gold
